# Are men who have sex with men in Europe protected from hepatitis B?

**DOI:** 10.1017/S0950268820000163

**Published:** 2020-02-13

**Authors:** M. Brandl, A. J. Schmidt, U. Marcus, M. an der Heiden, S. Dudareva

**Affiliations:** 1 Department of Infectious Disease Epidemiology, Robert Koch Institute, Berlin, Germany; 2 Sigma Research, London School of Hygiene & Tropical Medicine (LSHTM), London, UK

**Keywords:** Hepatitis B, sexually transmitted infections (STIs), vaccine preventable diseases

## Abstract

Hepatitis B vaccination is recommended for men who have sex with men (MSM) in many countries, but information on vaccine coverage is scarce. We studied hepatitis B vaccination programmes and coverage among MSM in Europe to guide prevention. From a large (*N* = 174 209) pan-European MSM survey (EMIS-2010), we used data on self-reported hepatitis B vaccination, age, education, settlement size and disclosure of the same-sex sexual orientation (‘outness’). We excluded participants with a history of hepatitis B. In multilevel (participants, countries) logistic regression models, we calculated adjusted odds ratios (aOR) with 95% confidence intervals (95% CI). We analysed data of 163 987 MSM in 38 European countries: 38.3% were ‘out’ to all or almost all, 56.4% reported vaccination against hepatitis B and 65.5% lived in countries with free recommended hepatitis B vaccination for MSM. In the final model the odds for being vaccinated increased with outness (‘out to all or almost all’: aOR 1.76, 95% CI 1.70–1.83 *vs.* ‘out to no one’) and with living in countries, where hepatitis B vaccination was recommended and free-of-charge for MSM (aOR 2.21, 95% CI 1.47–3.32 *vs.* ‘no or unclear recommendation’). To increase hepatitis B vaccination coverage among MSM, implementation of MSM-specific recommendations and improvement of the societal climate for MSM is needed.

## Introduction

Hepatitis B vaccines have been available since the early 1980s and in 1992 the World Health Assembly called for the inclusion of hepatitis B vaccination in all national vaccination guidelines [1]. Almost all European countries responded by setting up universal programmes for the general population including childhood vaccination and/or targeted programmes for indication groups like men who have sex with men (MSM) and people with frequently changing sexual partners [2]. Sexually transmitted infections with hepatitis B were common among MSM before vaccination programmes were introduced [3]. A systematic review from 2013 concluded that hepatitis B surface antigen (HBsAg) prevalence in MSM was on average 22 times higher than that in the general population [4]. A more recent review, published in 2018, calculated HBsAg prevalences among MSM between 0.0% and 1.4%, based on estimates from six European countries [5]. Although below 2%, which implies low prevalence, the authors still considered MSM a priority group for immunisation.

Many European countries have issued MSM-specific recommendations, typically including coverage of vaccination costs. However, vaccination coverage might be influenced not only by national vaccination guidelines but also by the ability of health care systems to identify and reach vulnerable populations, respectively the willingness of MSM to disclose the same-sex sexual contacts to a health care provider and to ask for vaccination. The circumstances allowing MSM to disclose their sexuality and other socio-demographic parameters are crucial for vaccine uptake [6]. Younger age, gay sexual identity and higher socioeconomic status are among the factors positively associated with hepatitis B vaccination coverage in MSM [7].

We analysed how national hepatitis B vaccination strategies and socio-demographic factors like age, educational level, settlement size and the disclosure of the same-sex sexual orientation affect hepatitis B vaccination coverage of MSM in Europe. With our results we want to help identify gaps in vaccination coverage and guide public health measures.

## Methods

For this paper we used data from following sources: EMIS-2010 provided data on vaccination coverage among MSM across 38 European countries. We also used four sociodemographic variables. We further collected information on vaccination programmes (universal and targeted) through online literature research and a short survey.

### Dataset

The 2010 European MSM Internet Survey (EMIS-2010) was an open-access online questionnaire which was available from 6 June to 31 August 2010. The survey reached 181 434 men in 46 countries [8]. It was a multi-lingual, pan-European, cross-sectional sexual health needs assessment for MSM, including measures of risk and precautionary behaviours, history of sexually transmitted infections and access to healthcare services [9]. Data collection and recruitment of participants for EMIS-2010 had the goal to capture a large and diverse sample of MSM across Europe [10].

The detailed survey development and methods of EMIS-2010 are described elsewhere [9]. We excluded men from countries with less than 100 qualifying cases, and men living in Oversea Departments, Territories and Collectivises of France, in British Overseas Territories and Crown Dependencies, or in Greenland (*n* = 446). Furthermore, we excluded cases who answered in more than one place in logically inconsistent ways (*n* = 6779). This resulted in a final dataset of 174 209 eligible participants living in 38 European countries.

### Hepatitis B immunisation status

Men participating in EMIS-2010 were asked ‘Have you been vaccinated against hepatitis B?’ and the following answers were possible:

1. No, I am naturally immune to hepatitis B (because I had it in the past)
2. No, and I don't know if I'm immune
3. Yes, and I completed the course of three shots of vaccine
4. Yes, but I did not complete the course of three shots of vaccine
5. Yes, but I did not respond to the vaccinations
6. I don't know

We combined the three options starting with ‘Yes', calling it ‘History of hepatitis B vaccination'. Men who answered ‘No, and I don't know if I'm immune’ (2), ‘I don't know’ (6), as well as missing answers, were coded as ‘No history of hepatitis B vaccination’. Men who answered ‘No, I am naturally immune to hepatitis B (because I had it in the past)’ (1) were excluded from further analyses, leading to an analytic sample of 163 987 participants. We performed a sensitivity analysis with missing answers and ‘I don't know’ excluded.

Four socio-demographic variables were used for analyses: age group (according to quartiles: <25 years (q1), 25–39 years (q2 + 3), 40 + years(q4)); educational level (low: ISCED 1 + 2 (International Standard Classification of Educational Degrees 1997), medium: ISCED 3 + 4, high: ISCED 5 + 6); settlement size (medium-sized and smaller settlements: <500 000 inhabitants, big to very big cities: 500 000 or more inhabitants) and outness, i.e. the disclosure of the own sexual orientation to friends, family members and work/study colleagues (out to no one, a few, less than half, more than half, all or almost all). The variables were chosen prior to initiation of analyses and decisions were based on factors associated with the outcome that were found in previous studies [7, 11].

EMIS-2010 was approved by the Research Ethics Committee of the University of Portsmouth, United Kingdom (REC application number 08/09:21).

### Vaccination programmes

We conducted a literature search on targeted vaccination programmes for hepatitis B in the 38 European countries included in EMIS-2010. Reports and studies that contained information on national hepatitis B vaccination recommendations for indication groups like MSM or people with frequently changing sexual partners were extracted. When available, we also collected data on year of implementation and how vaccination costs were met. Additionally, we created a short survey and sent it to public health authorities in each of the 38 EMIS countries. It consisted of three items: whether a vaccination recommendation for MSM or people with frequently changing sexual partners existed, with the option for year of implementation; how costs were met in case a vaccination recommendation existed; whether there was any other relevant information on the national hepatitis B vaccination programme for key populations.

We used data from all available sources that were published up to 2018 and grouped countries according to consistency of results: ‘Clear MSM-specific vaccination recommendation’ when there were more positive than negative data sources. ‘Unclear’ when there were as many positive as negative sources and ‘No vaccination recommendation for MSM’ when we found more negative than positive sources. We combined the latter two groups to ‘No or unclear vaccination recommendation for MSM’.

Taking into account vaccination costs, countries in group ‘Clear MSM-specific vaccination recommendation’ were assigned to ‘Free vaccination for MSM’, ‘Co-payment required’ or ‘Full out-of-pocket payment required’. We combined the latter two groups to ‘MSM-specific vaccination recommendation with payment’. This resulted in an outcome ‘MSM-specific vaccination recommendation’ with three values for analyses: ‘Free vaccination for MSM’; ‘MSM-specific vaccination recommendation with payment’ and ‘No or unclear vaccination recommendation for MSM’.

Through online research we also collected data on national vaccination guidelines for the general population. For each country we extracted information on existing and former universal vaccination programmes, year of implementation and age groups that were reached in the year 2010, when EMIS took place. For analyses we created the variable ‘General vaccination programme’ that included participants who were possibly vaccinated as infants, children or adolescents.

### Statistical analyses

We performed descriptive analyses at the country level for hepatitis B vaccination status, age group, educational level, settlement size and outness and reported frequencies and proportions. Categorical variables were compared by the *χ*^2^ test. A *P*-value < 0.05 was considered statistically significant.

Univariable and multilevel (participants, countries) logistic regression analyses with outcome ‘hepatitis B vaccination history’ and country of residence as group variable were conducted. Variables associated with the outcome in univariable analysis at *P*-value < 0.05 were included in multivariable models. Stepwise forward model selection with independent variables age group, educational level, settlement size, MSM-specific vaccination recommendation and general vaccination programme was applied in order to find the best fitting model. It was assessed by likelihood ratio tests and *P*-values < 0.05 were considered statistically significant. Total numbers, odds ratios (OR), 95% confidence intervals (95% CI) of OR and the intraclass correlation coefficient (ICC) were calculated. We computed additional models including interactions between variables, but chose a multivariable model without interactions for the presentation of results.

## Results

### Hepatitis B immunisation status

Overall, 44.7% of EMIS participants completed the full course of three doses (Table 1). Malta had the highest proportion of fully vaccinated participants (62.2%), followed by Austria (56.2%) and Switzerland (54.7%). Lowest proportions were found in Ukraine (12.8%), Serbia (13.5%) and Lithuania (14.6%). An incomplete course was reported by 6.8% of participants. The least common answer was ‘Yes, but I didn't respond to the vaccination’ (1.6%). Of all participants, 22.9% answered ‘No, I'm not vaccinated against hepatitis B and I don't know if I'm immune’. Countries with the highest proportion of not vaccinated men were Slovenia (45.7%), Lithuania (45.2%) and Slovakia (42.2%). The third most common answer was ‘I don't know’ or missing (18.1%). In some countries it was the leading answer with Belarus (47.7%), Moldova (45.3%) and Bulgaria (41.4%) on the top. In total, 5.9% of participants answered ‘No, I am naturally immune to hepatitis B (because I had it in the past)’. Results of self-reported vaccination rates on the country-level are shown in Table 1.

**Table 1.** Answers to question ‘Have you been vaccinated against hepatitis B?’ by country

	Natural immunity^a^	No immunisation^b^	Full immunisation^c^	Incomplete immunisation^d^	Non-responder^e^	Unknown^f^ + missings
Country	*n* (%)	*n* (%)	*n* (%)	*n* (%)	*n* (%)	*n* (%)
Austria	157 (3.8)	743 (18.2)	2294 (56.2)	299 (7.3)	37 (0.9)	555 (13.6)
Belarus	16 (4.4)	95 (25.9)	69 (18.8)	6 (1.6)	6 (1.6)	175 (47.7)
Belgium	293 (7.4)	784 (19.7)	1924 (48.3)	275 (6.9)	84 (2.1)	622 (15.6)
Bosnia & H.	12 (8.0)	40 (26.7)	35 (23.3)	3 (2.0)	2 (1.3)	58 (38.7)
Bulgaria	45 (4.3)	352 (34.0)	171 (16.5)	32 (3.1)	7 (0.7)	429 (41.4)
Croatia	18 (3.5)	190 (36.7)	155 (30.0)	28 (5.4)	5 (1.0)	121 (23.4)
Cyprus	11 (4.1)	60 (22.5)	117 (43.8)	17 (6.4)	4 (1.5)	58 (21.7)
Czech Republic	96 (4.0)	914 (38.1)	841 (35.0)	86 (3.6)	35 (1.5)	428 (17.8)
Denmark	89 (5.1)	665 (38.2)	571 (32.8)	103 (5.9)	10 (0.6)	304 (17.4)
Estonia	21 (3.5)	201 (33.8)	147 (24.8)	49 (8.2)	4 (0.7)	172 (29.0)
Finland	41 (2.0)	745 (36.8)	638 (31.5)	213 (10.5)	11 (0.5)	378 (18.7)
France	755 (6.8)	2251 (20.1)	4959 (44.4)	1170 (10.5)	308 (2.8)	1721 (15.4)
Germany	2738 (5.0)	10 644 (19.6)	28 453 (52.3)	3657 (6.7)	693 (1.3)	8202 (15.1)
Greece	178 (6.1)	673 (22.9)	1156 (39.3)	260 (8.8)	28 (0.9)	649 (22.0)
Hungary	58 (2.8)	816 (39.5)	650 (31.5)	131 (6.3)	8 (0.4)	404 (19.5)
Ireland	90 (4.1)	620 (28.2)	864 (39.4)	168 (7.7)	49 (2.2)	403 (18.4)
Italy	1353 (8.5)	3597 (22.5)	6315 (39.4)	715 (4.5)	253 (1.6)	3751 (23.5)
Latvia	62 (8.8)	272 (38.4)	193 (27.3)	37 (5.2)	3 (0.4)	141 (19.9)
Lithuania	33 (5.6)	269 (45.2)	87 (14.6)	29 (4.9)	2 (0.3)	175 (29.4)
Luxembourg	14 (5.0)	54 (19.3)	135 (48.2)	24 (8.6)	6 (2.1)	47 (16.8)
Malta	2 (1.7)	24 (20.2)	74 (62.2)	5 (4.2)	0 (0.0)	14 (11.7)
Moldova	7 (6.0)	26 (22.2)	23 (19.7)	1 (0.8)	7 (6.0)	53 (45.3)
Netherlands	435 (11.5)	648 (17.1)	2052 (54.2)	315 (8.3)	57 (1.5)	280 (7.4)
N. Macedonia	7 (6.0)	30 (25.6)	36 (30.8)	2 (1.7)	4 (3.4)	38 (32.5)
Norway	76 (3.6)	474 (22.6)	716 (34.2)	259 (12.4)	19 (0.9)	552 (26.3)
Poland	129 (4.7)	905 (32.9)	1103 (40.2)	191 (7.0)	12 (0.4)	406 (14.8)
Portugal	297 (5.7)	848 (16.3)	2774 (53.5)	311 (6.0)	103 (2.0)	854 (16.5)
Romania	127 (5.5)	759 (32.6)	504 (21.6)	95 (4.1)	33 (1.4)	809 (34.8)
Russia	309 (6.1)	1444 (28.7)	1267 (25.2)	276 (5.5)	90 (1.8)	1649 (32.7)
Serbia	65 (5.9)	429 (38.8)	149 (13.5)	31 (2.8)	9 (0.8)	423 (38.2)
Slovakia	24 (4.1)	247 (42.2)	146 (24.9)	16 (2.7)	9 (1.5)	144 (24.6)
Slovenia	30 (3.0)	452 (45.7)	256 (25.9)	35 (3.5)	4 (0.4)	213 (21.5)
Spain	998 (7.6)	3046 (23.2)	5395 (41.2)	817 (6.2)	134 (1.0)	2721 (20.8)
Sweden	113 (3.6)	931 (29.7)	1088 (34.7)	322 (10.3)	18 (0.6)	660 (20.1)
Switzerland	293 (5.8)	831 (16.5)	2751 (54.7)	310 (6.2)	90 (1.8)	753 (15.0)
Turkey	136 (7.5)	613 (33.9)	550 (30.5)	98 (5.4)	15 (0.8)	395 (21.9)
Ukraine	148 (8.6)	589 (34.4)	219 (12.8)	41 (2.4)	32 (1.9)	682 (39.9)
UK	946 (5.4)	3645 (20.6)	9002 (50.8)	1387 (7.8)	556 (3.1)	2182 (12.3)
EMIS-2010	10 222 (5.9)	39 926 (22.9)	77 879 (44.7)	11 814 (6.8)	2747 (1.6)	31 621 (18.1)

a ‘No, I am naturally immune to hepatitis B (because I had it in the past)’.

b ‘No, and I don't know if I'm immune’.

c ‘Yes, and I completed the course of three shots of vaccine’.

d ‘Yes, but I did not complete the course of three shots of vaccine’.

e ‘Yes, but I did not respond to the vaccines’.

f ‘I don't know’.

### Vaccination programmes

We identified three sources for targeted vaccination programmes: the European Centre for Disease Prevention and Control (ECDC) [12], the Vaccine European New Integrated Collaboration Effort (VENICE) [13] and the European Liver Patients Association (ELPA) [14]. For 18 countries, we found additional sources on their vaccination programmes for indication groups MSM or people with frequently changing sexual partners [15–32]. Furthermore, contact persons from 34 European countries answered our short survey. In 28 cases, they confirmed the results of collected data from literature research. Results are summarized in Table 2.

**Table 2.** Results of online literature search and short survey on targeted vaccination programmes for indication groups MSM or people with frequently changing sexual partners

Group	Country	Data source				
		ECDC (2010) [12]	VENICE (2009) [13]	ELPA (2017) [14]	Short survey (2018)	Additional sources (1993–2018) [15–32]
Countries with clear MSM-specific vaccination recommendation	Austria	p	–	p^o^	p^o^	p
	Belgium	p	p^o^	p^o^	(p)^c^	p
	Croatia	–	–	p^f^	p^f^	p
	Cyprus	p	p^f^	–	n	–
	Denmark	p	p^(f)^	p^(f)^	p^(f)^	p
	Estonia	n	p^o^	–	p^o^	–
	France	p	p^(f)^	p^c^	p^c^	p
	Germany	p	p^f^	p^f^	p^f^	p
	Greece	n	–	p^f^	p^f^	–
	Ireland	p	p^(f)^	–	p^f^	p
	Italy	p	p^f^	p^f^	p^f^	p
	Luxembourg	n	p^(f)^	–	p^f^	–
	Netherlands	p	p^f^	p^f^	p^f^	p
	N. Macedonia	–	–	p^o^	p^o^	–
	Norway	p	p^f^	–	p^f^	–
	Slovenia	n	p^(f)^	p^f^	p^f^	–
	Spain	p	p^f^	n	p^f^	p
	Sweden	p	p^(f)^	n	p^c^	–
	Switzerland	–	–	–	P^c^	p
	Turkey	–	–	n	p^c^	p
	UK	p	p^f^	p^f^	p^f^	p
Unclear countries	Belarus	–	–	–	–	–
	Bosnia & H.	–	–	p^o^	n	–
	Bulgaria	p	p^o^	n	–	n
	Hungary	n	p^f^	p^o^	n	–
	Slovakia	n	p^f^	n	p^f^	–
Countries with no vaccination recommendation for MSM	Czech Rep.	–	–	–	n	–
	Finland	n	n	p^c^	(p)^f^	(p)
	Latvia	n	n	–	n	–
	Lithuania	n	n	–	n	–
	Malta	n	n	–	p^o^	–
	Moldova	–	–	–	(p)^f^	–
	Poland	n	n	n	p^o^	n
	Portugal	n	n	p^c^	–	n
	Romania	n	–	p^o^	n	n
	Russia	–	–	–	n	–
	Serbia	–	–	n	–	–
	Ukraine	–	–	n	n	–

ECDC, European Centre for Disease Prevention and Control; ELPA, European Liver Patients' Association; MSM, Men who have sex with men; VENICE, Vaccine European New Integrated Collaboration Effort.

n = no MSM-specific vaccination recommendation; p = MSM-specific vaccination recommendation; (p) = MSM-specific vaccination recommendation implemented after the year 2010, treated like no MSM-specific vaccination recommendation; – = no information.

^c^ = co-payment required; ^f^ = free vaccination; ^(f)^ = free vaccination for some recipients; ^o^ = full out-of-pocket payment.

In 21 of 38 European countries clear vaccination recommendations for MSM or people with frequently changing sexual partners were in place, 12 countries had no vaccination recommendations for MSM and in five countries the situation was unclear. These 17 countries together were coded ‘No or unclear vaccination recommendation for MSM’ for analysis.

**Fig. 1.**
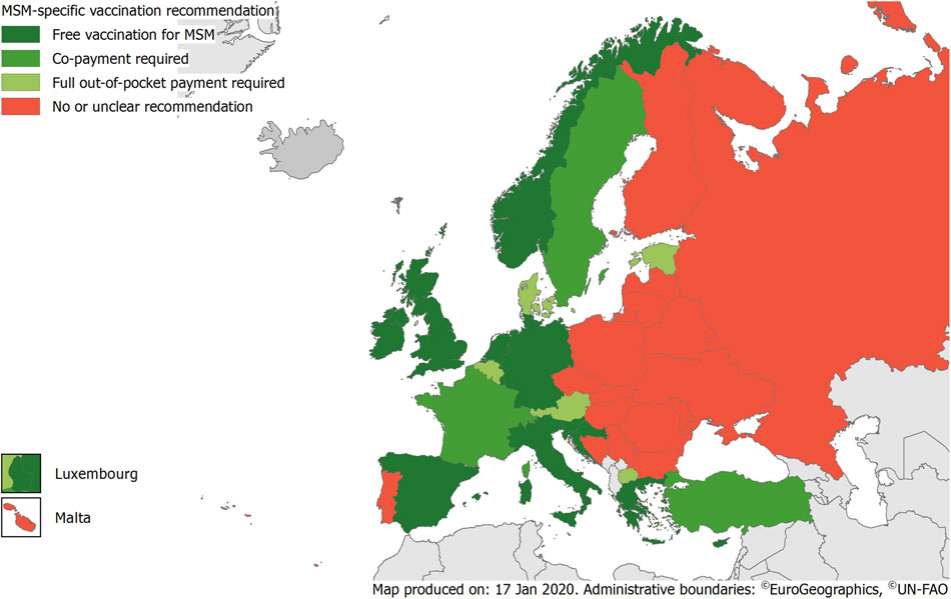
Map of Europe showing the classification of all 38 countries regarding their MSM-specific vaccination recommendations.

ELPA, VENICE and our short survey contained additional information on the way how vaccination costs were met for the 21 countries with clear recommendations. Figure 1 shows a map with the classification of all 38 countries according to combined data.

Identified data sources for general vaccination programmes were a review by Lernout *et al*. [2] and the websites of ECDC and the World Health Organization (WHO). Only 6 of 38 investigated countries (Denmark, Finland, the Netherland, Norway, Sweden and the UK) had not yet implemented universal hepatitis B vaccination programmes in 2010, when EMIS data was collected (Supplementary Table S1). In 29 countries universal programmes for infants or new-borns were in place, in three countries for adolescents or children. Additional programmes for adolescents, adults or children had been active in 12 countries. Due to the age of the 163 987 participants in the final dataset, only 20 660 (12.6%) men were possibly affected by universal vaccination programmes.

### Statistical analyses

In the sample included in further analysis (*n* = 163 987), that does not contain men who were naturally immune, 92 440 (56.4%) reported a positive vaccination history and 71 547 (43.6%) reported no history of vaccination. Their characteristics regarding age group, educational level, settlement size, outness, MSM vaccination recommendation and general vaccination programme are displayed in Table 3.

**Table 3.** Characteristics of EMIS participants by hepatitis B vaccination history

Characteristics	Total (N = 163 987)	Hepatitis B vaccination history	
		Yes (*n* = 92 440)	No (*n* = 71 547)
	*N* (%)	*n* (%)	*n* (%)
Age group:			
< 25	40 211 (24.5)	21 818 (23.6)	18 393 (25.7)^a^
25–39	81 750 (49.9)	46 629 (50.4)	35 121 (49.1)
40 +	42 026 (25.6)	23 993 (26.0)	18 033 (25.2)
Educational level (6 levels of ISCED^b^):			
Low (ISCED 1,2)	13 002 (8.0)	6294 (6.9)	6708 (9.5)
Medium (ISCED 3,4)	68 705 (42.2)	37 998 (41.3)	30 707 (43.4)
High (ISCED 5,6)	81 004 (49.8)	47 657 (51.8)	33 347 (47.1)
Settlement size (number of inhabitants):			
Medium-sized or smaller settlements (< 500 000)	87 972 (54.9)	47 790 (52.9)	40 182 (57.6)
Big to very big cities (500 000+)	72 167 (45.1)	42 620 (47.1)	29 547 (42.4)
Outness (being ‘out' to family/friends/work or study colleagues):			
No one	17 245 (10.6)	7684 (8.4)	9561 (13.5)
Few	34 992 (21.4)	16 725 (18.2)	18 267 (25.7)
Less than half	17 793 (10.9)	9584 (10.4)	8209 (11.5)
More than half	30 636 (18.8)	17 992 (19.6)	12 644 (17.8)
All or almost all	62 435 (38.3)	40 025 (43.5)	22 410 (31.5)
MSM-specific vaccination recommendation:			
No or unclear recommendation	26 812 (16.3)	10 816 (11.7)	15 996 (22.4)
Payment required^c^	29 787 (18.2)	17 518 (18.9)	12 269 (17.1)
Free vaccination	107 388 (65.5)	64 106 (69.4)	43 282 (60.5)
General vaccination programme:			
Not affected age groups	143 327 (87.4)	80 278 (86.8)	63 049 (88.1)
Age groups reached	20 660 (12.6)	12 162 (13.2)	8498 (11.9)

ISCED, International Standardised Classification of Educational Degrees; MSM, Men who have sex with men.

a *χ*^2^ test: *P* < 0.001 for all variables.

b Six levels of the ISCED, 1997 version.

c Co-payment or full out-of-pocket payment required.

### Multivariable analysis

The outcome ‘vaccination history’ was positively associated with age between 25 and 39 years, higher educational levels, living in big to very big cities and increasing outness (Table 4). In countries that had an MSM vaccination recommendation in place, chances for participants to have positive vaccination histories were about doubled for vaccination with payment (OR 1.96, 95% CI 1.26–3.06) and for free vaccination (OR 2.21, 95% CI 1.47–3.32), without statistically significant difference. Men in age groups that could have been affected by universal vaccinations also had increased chances to have a positive vaccination history (OR 1.68, 95% CI 1.61–1.75).

**Table 4.** Univariable and multivariable analysis of hepatitis B vaccination history

Variable	Value	Crude OR^a^ (95% CI)	Adjusted OR^b^ (95% CI) *n* = 158 206
Age group	<25	0.89 (0.87–0.92)^c^	0.94 (0.91–0.96)
	25–39	ref.	ref.
	40+	1.00 (0.98–1.03)^d^	0.94 (0.92–0.96)
Educational level	Low (ISCED^e^ 1,2)	ref.	ref.
	Medium (ISCED 3,4)	1.32 (1.27–1.37)	1.37 (1.32–1.43)
	High (ISCED 5,6)	1.52 (1.47–1.58)	1.81 (1.74–1.89)
Settlement size (number of inhabitants)	Medium-sized or smaller settlements (< 500 000)	ref.	ref.
	Big to very big cities (500 000+)	1.21 (1.19–1.24)	1.23 (1.21–1.26)
Outness (being ‘out’ to family/friends/work or study colleagues)	No one	ref.	ref.
	Few	1.14 (1.10–1.18)	1.16 (1.11–1.20)
	Less than half	1.45 (1.39–1.52)	1.31 (1.25–1.37)
	More than half	1.77 (1.71–1.84)	1.50 (1.44–1.56)
	All or almost all	2.22 (2.15–2.30)	1.76 (1.70–1.83)
MSM-specific vaccination recommendation	No or unclear recommendation	ref.	ref.
	Payment required^f^	2.11 (2.04–2.18)	1.96 (1.26–3.06)^g^
	Free vaccination for MSM	2.19 (2.13–2.25)	2.21 (1.47–3.32)
General vaccination programme	Not affected age groups	ref.	ref.
	Age groups reached	1.12 (1.09–1.16)	1.68 (1.61–1.75)

CI, confidence interval; ISCED, International Standardised Classification of Educational Degrees; MSM, Men who have sex with men; OR, odds ratio; ref, reference.

a Univariable logistic regression.

b Multilevel, multi-variable logistic regression with two levels (participants, countries).

c All *P*-values < 0.001 except when marked otherwise.

d *P*-value 0.860.

e Six levels of the ISCED, 1997 version.

f Co-payment or full out-of-pocket payment required.

g *P*-value 0.003.

The random effect of countries was measured with the ICC and was responsible for about 8% of the total variance of the model (ICC = 0.08, 95% CI 0.05–0.12). We ran a sensitivity analysis excluding missing answers and participants who answered ‘I don't know’ which did not alter the magnitude or direction of association much for most variables besides age group (<25: OR 1.39, 95% CI 1.34–1.44) and general vaccination programme (age groups reached: OR 2.87, 95% CI 2.71–3.03). Full results for sensitivity analysis can be found in Supplementary Table S2.

## Discussion

We analysed the answers from a large dataset of MSM living in Europe to determine what factors were associated with hepatitis B vaccination coverage. Two country-level (MSM-specific vaccination recommendations and universal vaccination programmes) and four individual-level (age, educational level, settlement size and outness) covariates showed associations with the outcome across descriptive, univariable and multivariable analyses, providing evidence for targeted public health actions.

Overall, 56.4% of participants had a positive history of vaccination in descriptive analysis. Data on hepatitis B vaccination coverage among MSM in Europe are scarce and we only found a few national studies for comparison. Estimates that were based on serological proof ranged from 14% in a Danish study [33] to 30–38% in a Dutch study [34]. Another Dutch study, however, found a self-reported vaccination rate of 50% [35], which is similar to our results for the Netherlands (54.4%). In the Danish study participants also reported their vaccination rates, which were 18% higher than results from blood tests showed [33]. We concluded that our results are prone to recall bias and probably overestimate actual vaccination rates. Confusion of vaccination against hepatitis B with hepatitis A and a lack of knowledge about hepatitis could be responsible for this effect.

Contrary to previous studies on MSM and factors that influence hepatitis B vaccination, we found a negative association between younger age and the outcome [7]. In multivariable analysis, however, men who could have been affected by universal vaccination programmes had higher chances to have a positive vaccination history and the effect was of similar magnitude as that of MSM-specific vaccination recommendations. We found that MSM with higher educational levels, who are living in bigger settlements, and are out about their sexual orientation had higher odds to be vaccinated against Hepatitis B. MSM who are better educated and those living in big cities have easier access to health care systems and gay-friendly health care providers in many countries. Outness facilitates MSM to accept recommendations that are directed specifically at them. An analysis with interactions revealed that outness had an impact on vaccination history especially in older participants and younger men were vaccinated regardless of their outness. This is probably explained by universal vaccination programmes and the time of coming out.

Previous studies already showed that homonegative social and legal climates obstructed access to prevention services and lead to lower levels of precautionary behaviour regarding sexually transmitted infections like HIV [36]. MSM in high-stigma countries lacked control over possible infections and were therefore more vulnerable towards HIV [6]. With this study we could show that the same might be true for hepatitis B. The consequence for public health and political measures should be to reduce both stigmatisation of LGBTI (lesbian, gay, bisexual, trans and intersex) citizens and to reinforce vaccinations for target populations.

One of the strengths of our study is that we combined data from various sources, extended by results from a self-provided short survey, to measure the influence of hepatitis B vaccine recommendations on the vaccination rates of MSM in Europe. We were able to use a variable on national vaccination recommendations that is backed up by a maximum of evidence. While VENICE [13] and ELPA [14] named MSM specifically in their reports, the ECDC [12] report named ‘multiple sex partners’ as key population. Our decision to use these key populations with equal value was backed up by a high concordance between these three reports and by findings from additional sources. The reports from VENICE and ECDC were published around the same time as EMIS took place. The report by ELPA on the other hand was published in 2016 and changes in vaccination guidelines could have happened in-between. However, results concurred largely with data from our other sources and only for Finland we found proof for implementation after the year 2010 [29].

For some countries like Malta, Poland and Romania most sources negated a vaccination recommendation for MSM with the exception of one source, respectively, that attested a recommendation with full out-of-pocket payment for the recipient. It is possible that these are two views of the same guideline by different health care professionals. The response from Russia to the short survey highlighted another difficulty in vaccination guideline interpretation. Since 2001 a national programme offered free vaccination against hepatitis B for all persons under the age of 55 years. In countries where no specific programme for MSM exists, but vaccination is recommended and available for everyone free of charge, it remains unclear if target populations are more encouraged to get vaccinated. Not naming a highly stigmatised indication group like MSM in vaccination recommendations might even be beneficial for their vaccination rates [37].

Our synthesised results imply that 17 countries had either clearly no recommendation for MSM or uncertainties regarding their vaccination guidelines. Fourteen of these countries were in the Eastern part of Europe, including Baltic countries, where vaccination rates were historically low [38]. It is possible that underlying regional effects are in part responsible for the effect that was attributed to vaccination recommendations and programmes in our analyses.

Participants from 10 European countries reported vaccination rates above the EMIS average. Eight of these countries had implemented MSM-specific vaccination recommendations. Malta and Portugal are the two exceptions and their high rates despite a lack of targeted programmes might be due to homopositive climates facilitating access to health services [39] and (in case of Malta) the inherited system of genitourinary medicine clinics, which appears highly effective for delivering quality sexual health services [40].

Only about 20 660 (12.6%) participants from 18 countries might have been vaccinated by a general vaccination programme for infants or adolescents. Most of them (15 891) were from Italy, Spain, Portugal and France, and their maximum age was 31 years. Of these men, 58.9% reported a positive vaccination history which was only slightly higher compared to remaining men in the dataset (56.0%). This might be due to a lack of recall of childhood vaccination. We recoded the item ‘No, and I don't know if I'm immune’ to ‘No vaccination history’. Our hypothesis was that of men who answered ‘I don't know’ the majority actually didn't receive a hepatitis B vaccination, they were true-negatives and vulnerable. Men reached by a universal vaccination programme or a catch-up programme against hepatitis B in their childhood and are not aware of it were recoded to ‘No vaccination history’ as well. That means there could have been false-negatives who in fact were protected. Thus, the true effect of universal programmes could be higher than our results imply. Sensitivity analysis excluding men who didn't know their vaccination status and missings supported this assumption.

### Limitations

Information on vaccination recommendations for key populations was collected in all three examined reports and our own short survey through questionnaires. Despite differences and insufficiencies in the 22 sources used, we rated the respective data as equally valid. Inconsistencies in findings may be due to the fact that the answers of contact persons rely on information provided by different specialists. The number of sources we used is also not exhaustive. More national guidelines or international reports on vaccination programmes might have altered assignments of single countries.

As EMIS was based on an online questionnaire only self-reported vaccination rates could be analysed in this study. True vaccination rates may be higher though, especially in younger age groups, because they might have been vaccinated in early years of their life and just were not aware of it. Vaccination rates and all other variables derived from the EMIS-2010 dataset were self-reported and thereby prone to recall bias, which could also lead to an overestimation of actual vaccination rates. Additionally, EMIS participants might have e.g. confused hepatitis A and B, for both of which vaccinations exist. However, questions on hepatitis A vaccination status or on knowledge about hepatitis were not part of EMIS-2010.

The data analysed in this study are from 2010, and coverage and associated factors may therefore have changed since then. However, the follow-up EMIS-2017 data suggest that overall there has been no substantial increase in vaccine uptake since EMIS-2010 [41].

## Conclusions

We provide the latest and most complete update on hepatitis B vaccination recommendations for MSM in Europe. Differences in national vaccination guidelines across European countries affect vaccination rates and MSM in many parts of Europe are still vulnerable to hepatitis B, even though vaccines are a widely accessible way of protection. However, men who live in countries with MSM-specific vaccination recommendations and homopositive societies have higher chances of being vaccinated against hepatitis B.

### Recommendations

Vaccination recommendations that target MSM specifically are essential to increase vaccination coverage. The societal climate for LGBTI citizens in Europe facilitating MSM to be out about their sexual orientation should be further improved to enable appropriate access to MSM-specific health care services. A unified European approach is crucial in battling stigmata that persist towards both sexually transmitted infections and sexual minorities, in order to protect these vulnerable populations from disease through united Public Health measures. Further steps to increase vaccine uptake among MSM should be investigated.

## Supplementary material

For supplementary material accompanying this paper visit https://doi.org/10.1017/S0950268820000163. click here to view supplementary material
