# Supplementary material for: Are men who have sex with men in Europe protected from hepatitis B?
Source: Epidemiol Infect. 2020 Feb 13;148:e27. doi: 10.1017/S0950268820000163 (PMC7026898; doi:10.1017/S0950268820000163)
Supplement: Supplementary file 1 [file S0950268820000163sup001.docx]

**Supplementary material**

Supplementary Table S1: Universal vaccination programmes with year of implementation plus additional programmes with years they were effective and age groups covered

| Country | Data Sources: Lernout et al. [2], adapted; ECDC; WHO. | | | | |
| --- | --- | --- | --- | --- | --- |
|  | Universal programme (national level) | Year of intro-duction | Additional universal programme | Years effective | Age groups covered in 2010^a^ |
|  | | | | | |
| Austria | Infant | **1997** |  |  | 0-13 |
| Belarus | New-born | **1999** | Adolescent (13 y) | 1999-2004 | 0-11/**19-24** |
| Belgium | Infant | **1999** | Adolescent (12 y) | 1999-2011 | **0-24** |
| Bosnia & H. | New-born | **2001** |  |  | 0-9 |
| Bulgaria | New-born | **1991** |  |  | **0-19** |
| Croatia | Infant | **2007** | Adolescent (12-13 y) | 1999-2009 | 0-3 / **14-24** |
| Cyprus | Infant | **1990** |  |  | **0-20** |
| Czech Republic | Infant | **2001** |  |  | 0-9 |
| Denmark | No universal program |  |  |  | None |
| Estonia | New-born | **2003** | Adolescent (12-13 y) | 1999-2012 | 0-7/**12-24** |
| Finland | No universal program |  |  |  | None |
| France | Infant | **1995** | Adolescent (11-13 y) | 1994-1999 | **0-16**/**24-29** |
| Germany | Infant | **1995** |  |  | **0-16** |
| Greece | Infant | **1998** |  |  | 0-12 |
| Hungary | Adolescent (14 y) | **1999** |  |  | **14-25** |
| Ireland | Infant | **2008** |  |  | 0-2 |
| Italy | Infant | **1991** | Adolescent (12 y) | 1991-2003 | **0-31** |
| Latvia | Infant | **1997** |  |  | 0-13 |
| Lithuania | New-born | **1998** |  |  | **0-14** |
| Luxembourg | Infant | **1996** |  |  | 0-13 |
| Malta | Infant | **1997** |  |  | 0-13 |
| Moldova | New-born | **1995** | Adolescent (10-14 y) | 2005 | **0-19** |
| Netherlands | Infant | 2011 |  |  | None |
| North Macedonia | New-born | **2004** |  |  | 0-6 |
| Norway | Infant | 2016 |  |  | None |
| Poland | New-born | **1996** | Adolescent (14 y) | 2000-2008 | **0-14**/**16-24** |
| Portugal | New-born | **2000** | Adolescent (10-13 y) | 1994-2011 | **0-29** |
| Romania | New-born | **1995** | Child (9 y)/Adults (18 y) | 1999-2007/2004-07 | **0-24** |
| Russia | New-born | **2002** |  |  | 0-8 |
| Serbia | New-born | **2005** |  |  | 0-5 |
| Slovakia | Infant | **1998** | Child (10 y) | 2004 | 0-12 |
| Slovenia | Child (5-6 y) | **1998** |  |  | **5-18** |
| Spain | New-born | **2003** | Adolescent (12 y) | 1996-2012 | 0-7/**10-26** |
| Sweden | Infant | 2011 |  |  | None |
| Switzerland | Adolescent (11 y) | **1998** |  |  | **11-23** |
| Turkey | Infant | **1998** |  |  | 0-12 |
| United Kingdom | Infant | 2017 |  |  | None |
| Ukraine | New-born | **2000** |  |  | 0-10 |

ECDC: European Centre for Disease Prevention and Control; WHO: World Health Organization; y: years old.

^a^ Marked in bold are age groups of men who participated in EMIS-2010 due to national laws on age of sexual consent.

Supplementary Table S2: Sensitivity analysis excluding unknown + missings

| **Variable** | **Value** | **Crude OR^a^ (95% CI)** | **Adjusted OR^b^ (95% CI)**  **n=128062** |
| --- | --- | --- | --- |
|  | | | |
| **Age group** | <25 | 1.47 (1.42-1.51)^c^ | 1.39 (1.34-1.44) |
|  | 25-39 | ref. | ref. |
|  | 40+ | 0.86 (0.84-0.89) | 0.83 (0.80-0.85) |
|  | | | |
| **Educational level** | Low (ISCED^d^ 1,2) | ref. | ref. |
|  | Medium (ISCED 3,4) | 1.22 (1.17-1.28) | 1.24 (1.18-1.30) |
|  | High (ISCED 5,6) | 1.20 (1.15-1.26) | 1.51 (1.43-1.59) |
|  | | | |
| **Settlement Size (number of inhabitants)** | Medium-sized or smaller settlements (<500000) | ref. | ref. |
|  | Big to very big cities (500000+) | 1.15 (1.12-1.18) | 1.23 (1.19-1.26) |
|  | | | |
| **Outness (Being ‘out’ to family/friends/work or study colleagues)** | No one | ref. | ref. |
|  | Few | 1.05 (1.00-1.10)^e^ | 1.10 (1.05-1.15) |
|  | Less than half | 1.28 (1.21-1.34)^f^ | 1.20 (1.14-1.27) |
|  | More than half | 1.55 (1.48-1.63) | 1.39 (1.32-1.46) |
|  | All or almost all | 1.88 (1.80-1.96) | 1.62 (1.55-1.69) |
|  | | | |
| **MSM-specific vaccination recommendation** | No or unclear recommendation | ref. | ref. |
|  | Payment required^g^ | 2.02 (1.94-2.10) | 1.99 (1.27-3.11)^h^ |
|  | Free vaccination for MSM | 2.16 (2.09-2.23) | 2.23 (1.48-3.34) |
|  | | | |
| **General vaccination**  **programme** | Not affected age groups | ref. | ref. |
|  | Age groups reached | 2.30 (2.20-2.40) | 2.87 (2.71-3.03) |

CI: Confidence Interval; ISCED (International Standardised Classification of Educational Degrees); MSM: Men who have sex with men; OR: Odds Ratio; ref: reference.

^a^ univariable logistic regression.

^b^ multilevel, multi-variable logistic regression with two levels (participants, countries).

^c^ all p-values < 0.001 except when marked otherwise.

^d^ six levels of the ISCED, 1997 version.

^e^ p-value 0.192.

^f^ p-value 0.028.

^g^ co-payment or full out-of-pocket payment required.

^h^ p-value 0.002.
